# Supplementary material for: The Owls Are Not What They Seem: Health, Mood, and Sleep Problems Reported by Morning and Evening Types with Atypical Timing of Weekend Sleep
Source: Clocks Sleep. 2025 Jul 11;7(3):35. doi: 10.3390/clockssleep7030035 (PMC12286085; doi:10.3390/clockssleep7030035)
Supplement: Supplementary file 1 [file clockssleep-07-00035-s001.zip › clockssleep-3595771-supplementary.pdf]

Supplementary Materials

The SIC (Single Item Chronotyping) in Russian (upper) and English (lower)

Publication in English: Putilov AA.,…, Mairesse O. Single-Item Chronotyping (SIC), a method to self-assess diurnal types by using 6 simple charts. Pers Ind Differ. 2021, 168:Article 110353) [6].

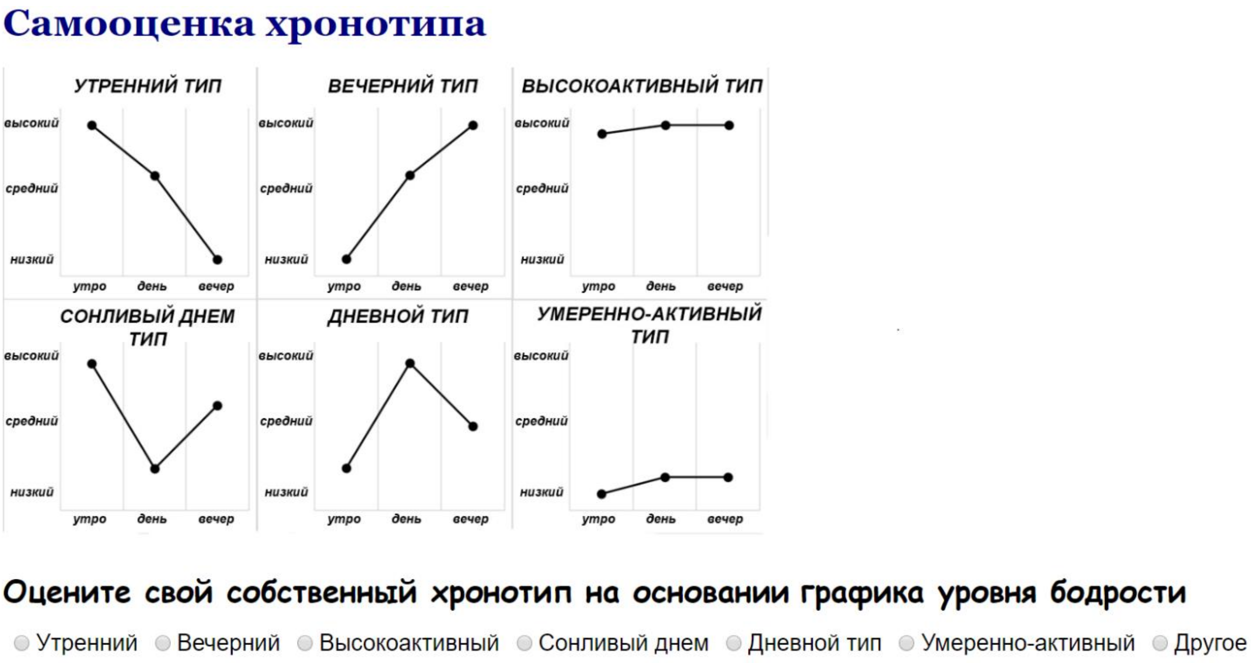

Self-assess your own chronotype by choosing one of six patterns of daily change in alertness level

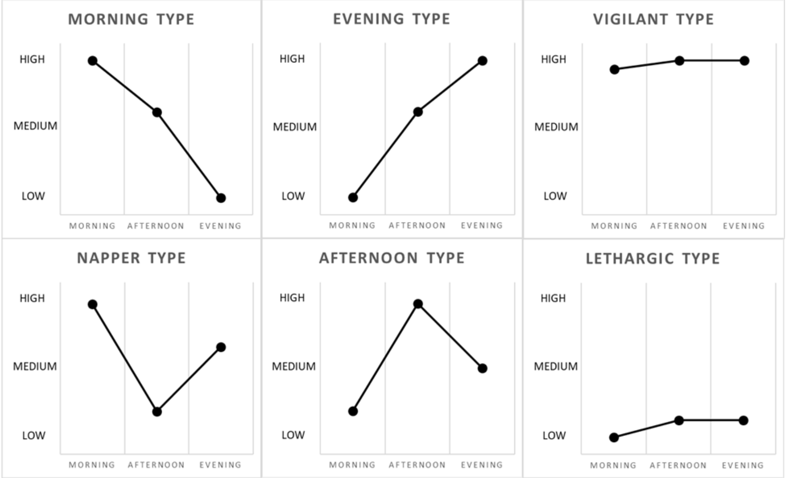

- Morning type: high level in the morning, middle in the afternoon, low in the evening
- Evening type: low level in the morning, middle in the afternoon, high in the evening
- Vigilant type: high level in the morning, high in the afternoon, high in the evening
- Lethargic type: low level in the morning, low in the afternoon, low in the evening
- Afternoon type: low level in the morning, high in the afternoon, low or middle in the evening
- Napper: high level in the morning, low in the afternoon, high or middle in the evening
- None of the above
